# Impact of NF-κB pathway on the apoptosis-inflammation-autophagy crosstalk in human degenerative nucleus pulposus cells

**DOI:** 10.18632/aging.102266

**Published:** 2019-09-13

**Authors:** Weiwei Yi, Yafeng Wen, Fuqiang Tan, Xi Liu, Haiyang Lan, He Ye, Bo Liu

**Affiliations:** 1 Department of Orthopaedic Surgery, The First Affiliated Hospital of Chongqing Medical University, Chongqing, China; 2 Department of Orthopaedic Surgery, Hechuan District People's Hospital of Chongqing, Chongqing, China; 3 Department of Orthopaedic Surgery, The Affiliated Hospital of Guizhou Medical University, Guizhou, China

**Keywords:** apoptosis, inflammation, autophagy, NF-κB pathway, nucleus pulposus cells

## Abstract

The NF-κB pathway has been reported to play a very important role in the process of intervertebral disc degeneration (IVDD). Our results demonstrated that knockdown of NF-κB with P65-siRNA can significantly decrease cell apoptosis and the expression of pro-inflammation factors TNF-α and IL-1β in LPS-induced nucleus pulposus cells (NPCs). However, the molecular mechanism of NF-κB pathway exerting anti-inflammation and anti-apoptosis function remains unclear. Some researchers reported that inhibiting NF-κB pathway can attenuate the catabolic effect by promoting autophagy during inflammatory conditions in rat nucleus pulposus cells. Therefore, we hypothesized that in human NPCs, inhibiting NF-κB pathway may also promote autophagy. Our results indicated that after knockdown of NF-κB, the autophagy was significantly increased and the expression of p-AKT and p-mTOR protein markedly decreased, but the level of autophagy was inhibited after treatment with AKT activator SC79, suggesting the involvement of AKT/mTOR–mediated autophagy was under autophagy activation. However, both LPS-induced NPCs apoptosis and expression of pro-inflammation factors were further increased by pretreatment with the autophagy inhibitor chloroquine (CQ). These suggested that inhibiting NF-κB pathway can promote autophagy and decrease apoptosis and inflammation response in LPS-induced NPCs. Meanwhile, autophagy triggered by NF-κB inhibition plays a protective role against apoptosis and inflammation.

## INTRODUCTION

Intervertebral disc (IVD) degeneration is one of the main causes of lower back pain (LBP), the incidence of which is getting higher and higher and has caused serious social problems [1]. Intervertebral disc degeneration (IVDD) is also a common pathological basis for most of the spine-related degenerative diseases, including intervertebral disc herniation, vertebral spondylolisthesis, spinal stenosis, nerve root pain and intervertebral discogenic low back pain [2]. Although the causes and molecular mechanisms of IVDD have not yet been fully elucidated, the loss of extracellular matrix, the excessive apoptosis of intervertebral disc cells and the inflammatory response play very important roles in the process of IVDD [3–5]. The production of excessive inflammatory factors can, on the one hand, inhibit the synthesis of extracellular matrix and, on the other hand, promote intervertebral disc cell apoptosis. The decreased number of intervertebral disc cells caused by excessive apoptosis of NPCs leads to a reduction in extracellular matrix synthesis, which further leads to disc degeneration. Consequently, the best treatment of IVDD is to inhibit the inflammatory response and excessive apoptosis of intervertebral disc cells.

Nuclear factor-kappaB (NF-κB) is a multifunctional transcription factor and is a member of the NF-κB / Rel proteins family. There are five subunits in mammals, but the most common form is p65/p50 dimer complex, which plays an important role in the inflammatory response, regulation of cell proliferation and apoptosis [6]. Typically, in unstimulated cells, NF-κB is usually present in the cytoplasm in an inactive form. When stimulated by various inflammatory factors, such as IL-1β, IL-6, TNF-α and LPS, NF-κB will be rapidly transferred to the nucleus and regulate target gene transcription and expression [7–8]. Many studies demonstrated that the expression of IL-1β, TNF-α, NF-κB and the number of apoptotic cells in degenerated intervertebral disc tissues were significantly higher than those in normal intervertebral disc tissues; in addition, their expression levels were positively correlated with the degree of intervertebral disc degeneration [9–10]. Jennewein et al. found that inhibiting the activity of NF-κB can reduce the apoptosis of malignant glioma cells [11]. Jieliang Shen et al. also found that overexpression SIRT1 in the nucleus pulposus cells can inhibit the activity of NF-κB and protect against cells apoptosis [12]. Therefore, we hypothesized that inhibiting NF-κB may reduce the expression of inflammatory factors and cell apoptosis in IVDD.

Autophagy is a conserved cellular process that eliminates damaged organelles and proteins and maintains cell self-stabilization state [13]. Many studies have also shown that autophagy plays a protective role in anti-apoptosis and anti-inflammation. Xu et al. found that in the rat intervertebral disc nucleus pulposus cells, inhibiting the activity of NF-κB can promote autophagy against intervertebral disc degeneration [14]. Up to now, the relationship between autophagy, apoptosis, inflammatory and the NF-κB pathway has not been reported in human NP cells. Therefore, we explored that whether in human intervertebral disc nucleus cells, inhibiting of NF-κB activity could promote autophagy against the apoptosis of nucleus pulposus cells and the expression of inflammatory factors.

## RESULTS

### Basal expression of TNF-α, IL-1β, P65 and cleaved caspase 3 in human NP tissues

**Figure 1:**
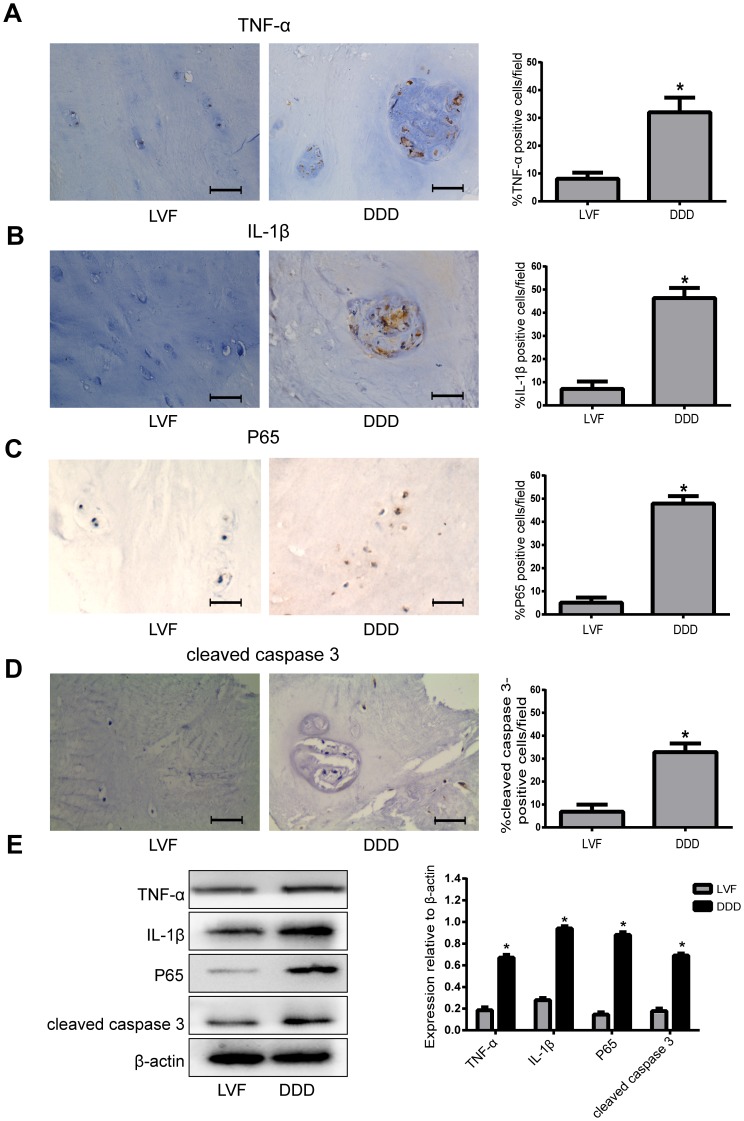
**The expression of TNF-α, IL-1β, P65 and cleaved caspase 3 in human NP tissues from LVF and DDD group.** (**A**), (**B**), (**C**) and (**D**) Immunohistochemistry results show that the levels of TNF-α (**A**), IL-1β (**B**), P65 (**C**) and cleaved caspase-3 (**D**) were significantly increased in NP tissues from patients with DDD (magnification ×400); (**E**) Western blot for the protein level of TNF-α, IL-1β, P65 and cleaved caspase 3. Values are means ±SEM.*p<0.05 vs. LVF group.

The expression levels of TNF-α, IL-1β, P65 and cleaved caspase 3 were significantly increased in the degenerative human NP tissues compared with the normal NP tissues as revealed by immunohistochemistry (Figure 1A–1D) and western blot (Figure 1E) analyses. These results suggested that NF-κB pathway may be closely related to the increase of inflammatory factors and apoptotic cells during the process of human IVDD.

### Effects of P65-siRNA and LPS treatment on human NPCs

**Figure 2:**
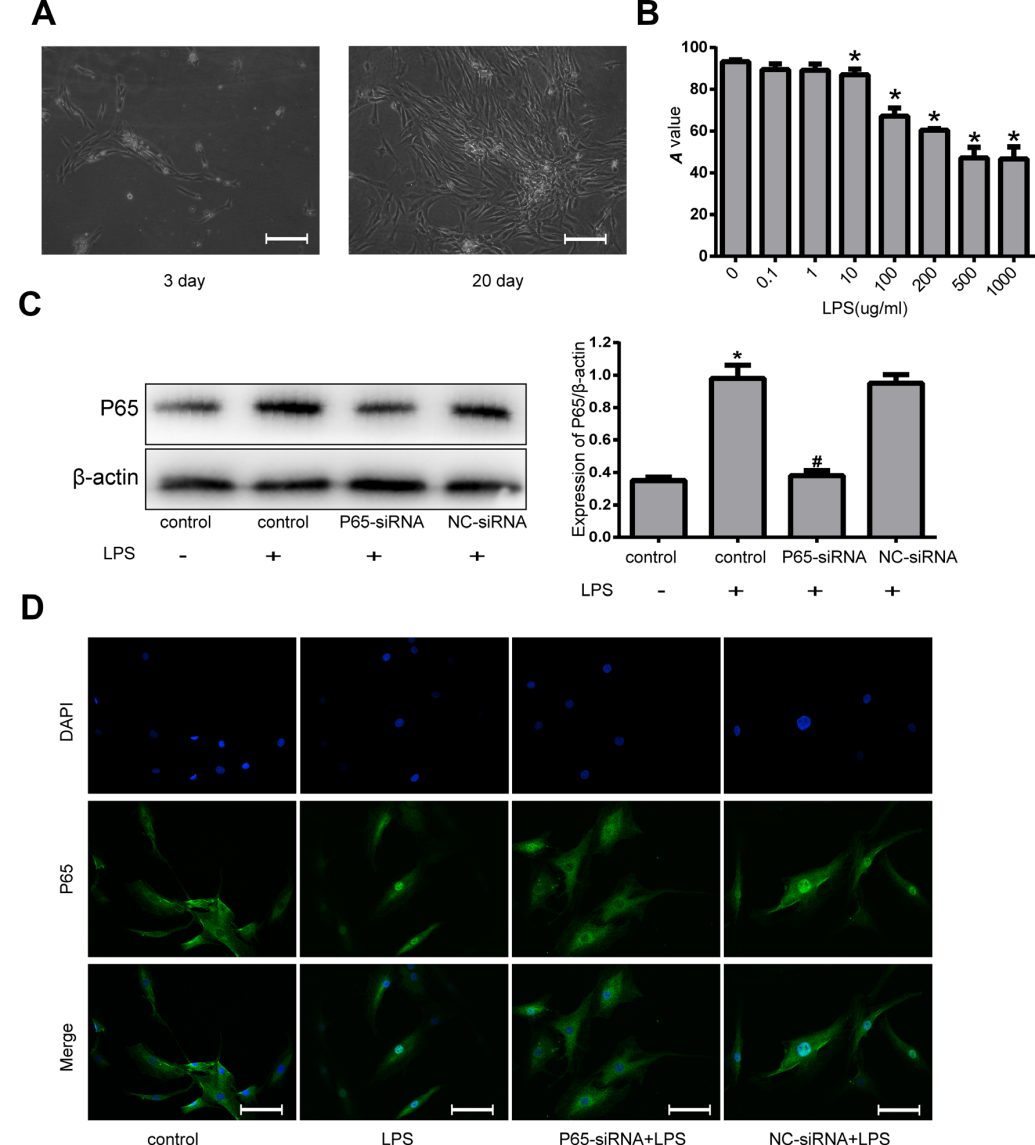
**Effects of NF-κB inhibition and LPS on human NPCs.** (**A**) The morphology of the primary NP cells was observed with inverted microscope (magnification ×200). (**B**) The viability of NPCs induced by various concentrations of LPS was measured by CCK-8. (**C**) Western blot analysis for the protein expression of P65. (**D**) The nuclear translocation of P65 was measured by Fluorescence immunocytochemistry (magnification ×200). Values are means ±SEM.*p<0.05 vs. control group, ^#^p<0.05 vs. LPS group.

As shown in Figure 2A, primary degenerated NP cells were mainly polygonal, irregular fusiform or flaming cell clusters. P0 generation nucleus pulposus cells grow slowly, taking more than 20 days to reach 80~90% confluence. In order to find the appropriate concentration of LPS to deal with NPCs, the Cell Counting Kit-8 (CCK-8) was used, the results of which showed that LPS significantly reduced the viability of NPCs. The group treated with 500 μg/ml or 1000 μg/ml LPS had the highest inhibition of viability of NPCs among any other concentrations of LPS, suggesting 500 μg/ml LPS may be the maximal effective dose for inhibiting the viability of NPCs (Figure 2B). Therefore, we decided to use 500 μg/ml LPS in the rest of the experiments in this study. To investigate the effects of P65-siRNA on NF-κB pathway, western blot was used to measure the protein expression of P65. The levels of P65 were markedly increased in NPCs stimulated with LPS compared with the control cells. However, the NPCs transfected with P65-siRNA markedly reduced the level of P65 (Figure 2C). Furthermore, the NPCs were subjected to immunofluorescence to analyze the nuclear translocation of P65. As shown in Figure 2D, knockdown of P65 inhibited the nuclear translocation of P65 compared with LPS treatment.

### Inhibiting NF-κB decreases apoptosis and inflammatory in LPS-induced NPCs

**Figure 3:**
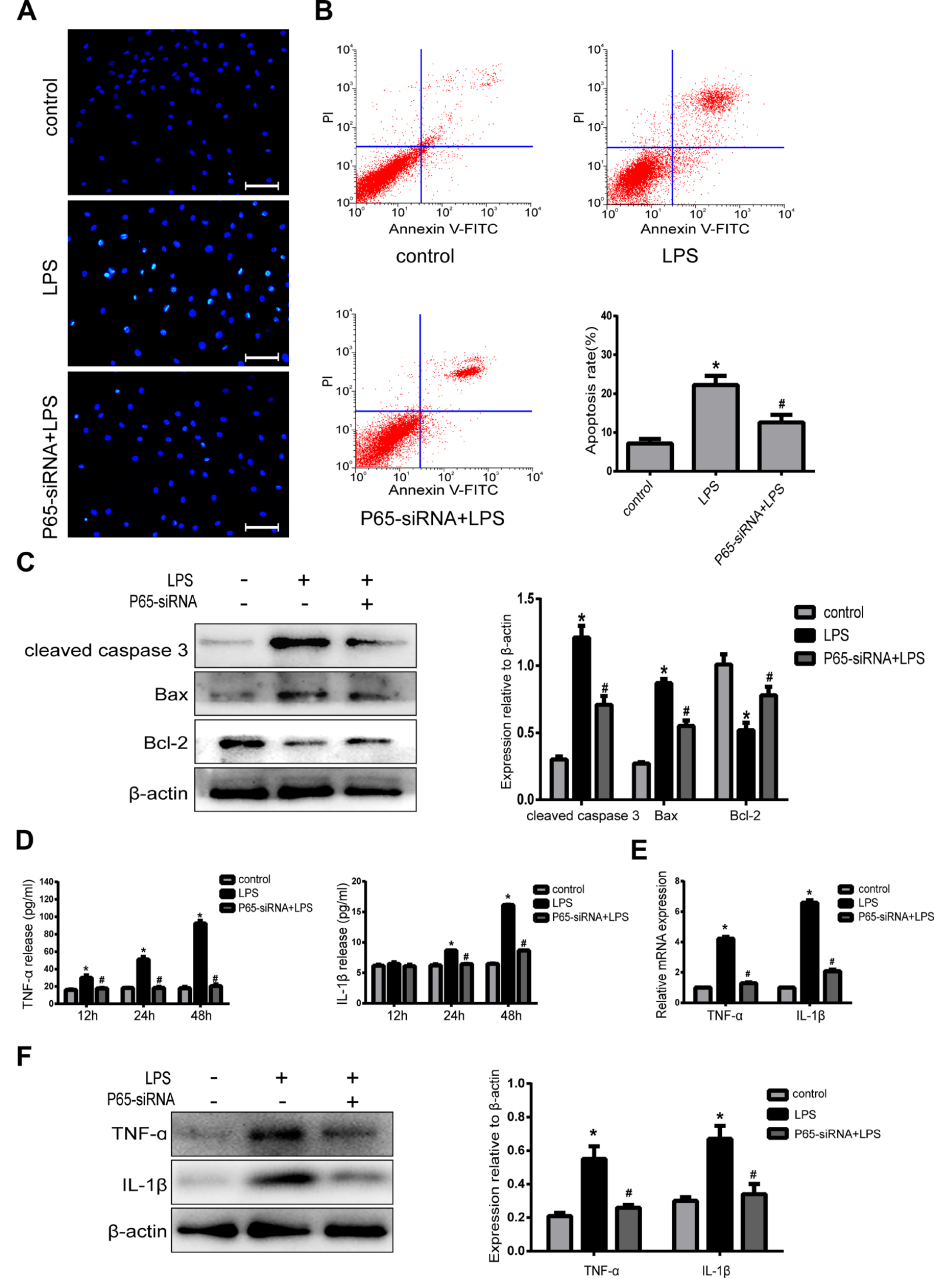
**Inhibiting NF-κB decreases apoptosis and inflammatory in LPS-induced NPCs.** (**A**) Morphologic changes in apoptotic NP cells were stained by Hoechst 33258 (Amplification ×200). (**B**) Apoptotic cells were stained with Annexin V-PE and PI, and analyzed by flow cytometry. (**C**) Western blot analysis for the protein expression of cleaved caspase 3, Bax and Bcl-2. (**D**) The expression of TNF-α and IL-1β in NPCs supernatant was measured by ELISA. (**E**) Real-time PCR analysis for the protein expression of TNF-α and IL-1β. (**F**) Western blot analysis for the protein expression of TNF-α and IL-1β. Values are means ±SEM.*p<0.05 vs. control group, ^#^p<0.05 vs. LPS group.

In order to demonstrate the association between the NF-κB pathway and NPCs apoptosis. Hoechst 33258 staining showed that there were less apoptotic cells with high bright fluorescent nuclei in P65-siRNA treatment group than LPS treatment group (Figure 3A). In addition, the results of Annexin V/PI double-staining demonstrated that the number of apoptotic NPCs was significantly decreased by P65-siRNA treatment compared with LPS treatment (Figure 3B). Simultaneously, western blot results showed that knockdown of P65 decreased the expressions of apoptotic protein cleaved caspase 3 and Bax; however, increased the expression of anti-apoptotic protein Bcl-2 compared with LPS treatment (Figure 3C). These experiments suggested that NF-κB inhibition decreased NP cell apoptosis in NP cells.

In order to further confirm the association between the NF-κB pathway and NPCs inflammation, the present study examined the effect of knockdown of P65 on LPS-induced proinflammatory factors. The ELISA results demonstrated that LPS treatment significantly increased the expression of TNF-α at 24h and IL-1β at 12h, 24h and both peaked at 48h compared with the control group. However, knockdown of P65 dramatically decreased the expression levels of TNF-α at 24h, 48h and IL-1β at 12h, 24h and 48h compared with LPS treatment (Figure 3D). In addition, real-time PCR (Figure 3E) and western blot (Figure 3F) results also demonstrated that knockdown of P65 markedly decreased the mRNA and protein expressions of TNF-α and IL-1β compared with LPS treatment. These experiments suggested that NF-κB inhibition reduces inflammation in NP cells.

### Inhibiting NF-κB promotes autophagy in LPS-induced NPCs

**Figure 4:**
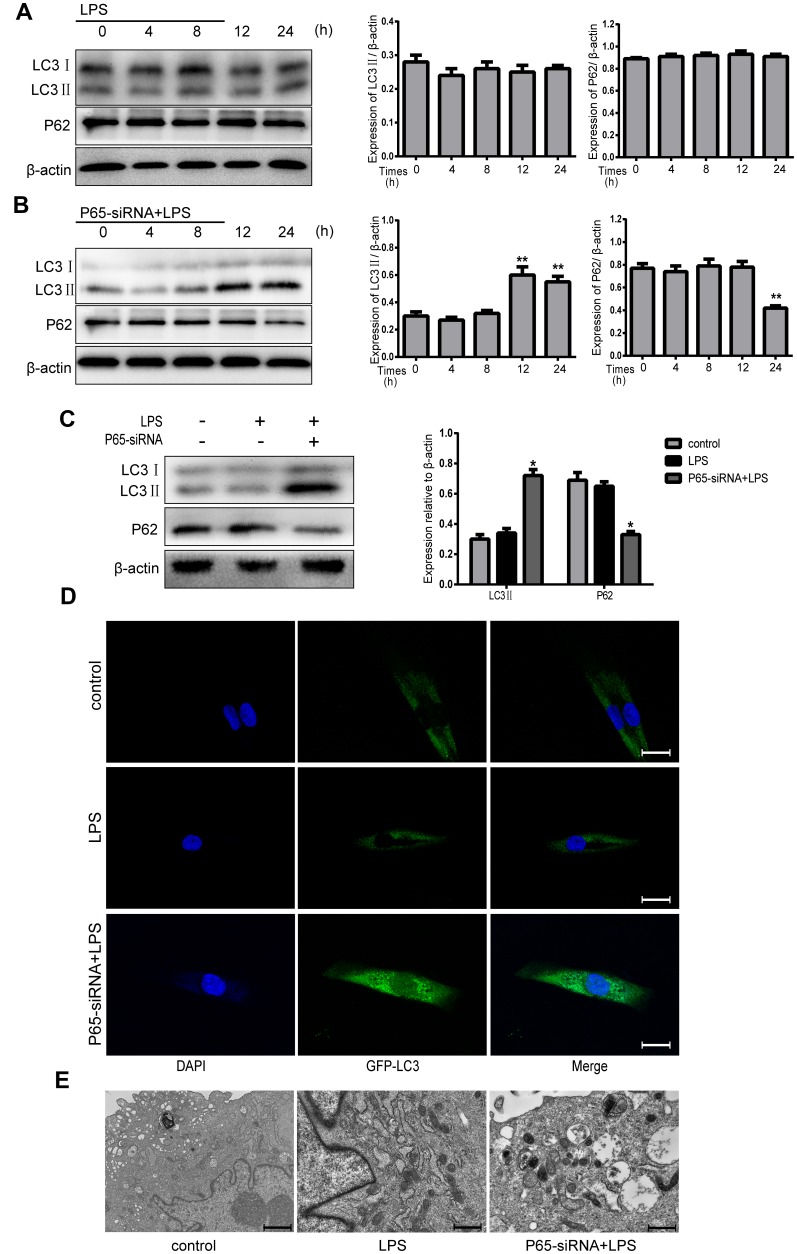
**Inhibiting NF-κB promotes autophagy in LPS-induced NPCs.** (**A**) After treatment with LPS for 4-24 h, the protein expression levels of LC3 II and P62 were measured by western blot. (**B**) After treatment with LPS plus p65-siRNA for 4-24 h, the protein expression levels of LC3 II and P62 were measured by western blot. (**C**) Western blot analysis for the protein expression levels of LC3 II and P62 after various treatment for 24 h. (**D**) NPCs were transfected with adenovirus containing GFP-LC3 and the formation and distribution of GFP-LC3 punctate were observed under confocal microscopy (Amplification×800). (**E**) Morphological observation of autophagy under transmission electron microscope (magnification ×30,000). Values are means ±SEM.*p<0.05 vs. control group, ^#^p<0.05 vs. LPS group, **p<0.05 vs. only p65-siRNA group.

In order to investigate the relationship between NF-κB pathway and autophagy in NPCs, the cells were treated with either LPS (500 ug/ml) or LPS plus P65-siRNA for 4, 8, 12 and 24 h. Western blot results revealed that treatment with LPS for 4-24h had no significant effect on the protein expression levels of LC3 II and P62, however the protein expression of LC3 II was significantly increased at 12h and 24h, and the protein expression of P62 was significantly decreased at 24h after treatment with LPS plus P65-siRNA (Figure 4A and 4B). To further confirm activation of autophagy, the time of drug treatment, 24h, was determined for the next experiment. Western blot results shown that treatment LPS plus P65-siRNA for 24h significantly increased the protein expression of LC3 II and decreased the protein expression of P62 compared with LPS treatment (Figure 4C). In addition, Fluorescence-based detection of LC-3 isoforms were directly observed in NP cells, knockdown of P65 resulted in a significant improvement in the LC3 puncta, indicative of increased autophagosome formation (Figure 4D). Furthermore, TEM observation found that the number of typical double-membraned autophagosomes in P65-siRNA treatment group was more than that in the LPS treatment group (Figure 4E).

### Inhibiting NF-κB blocks apoptosis and inflammation of degenerative human NP cells by promoting autophagy

**Figure 5:**
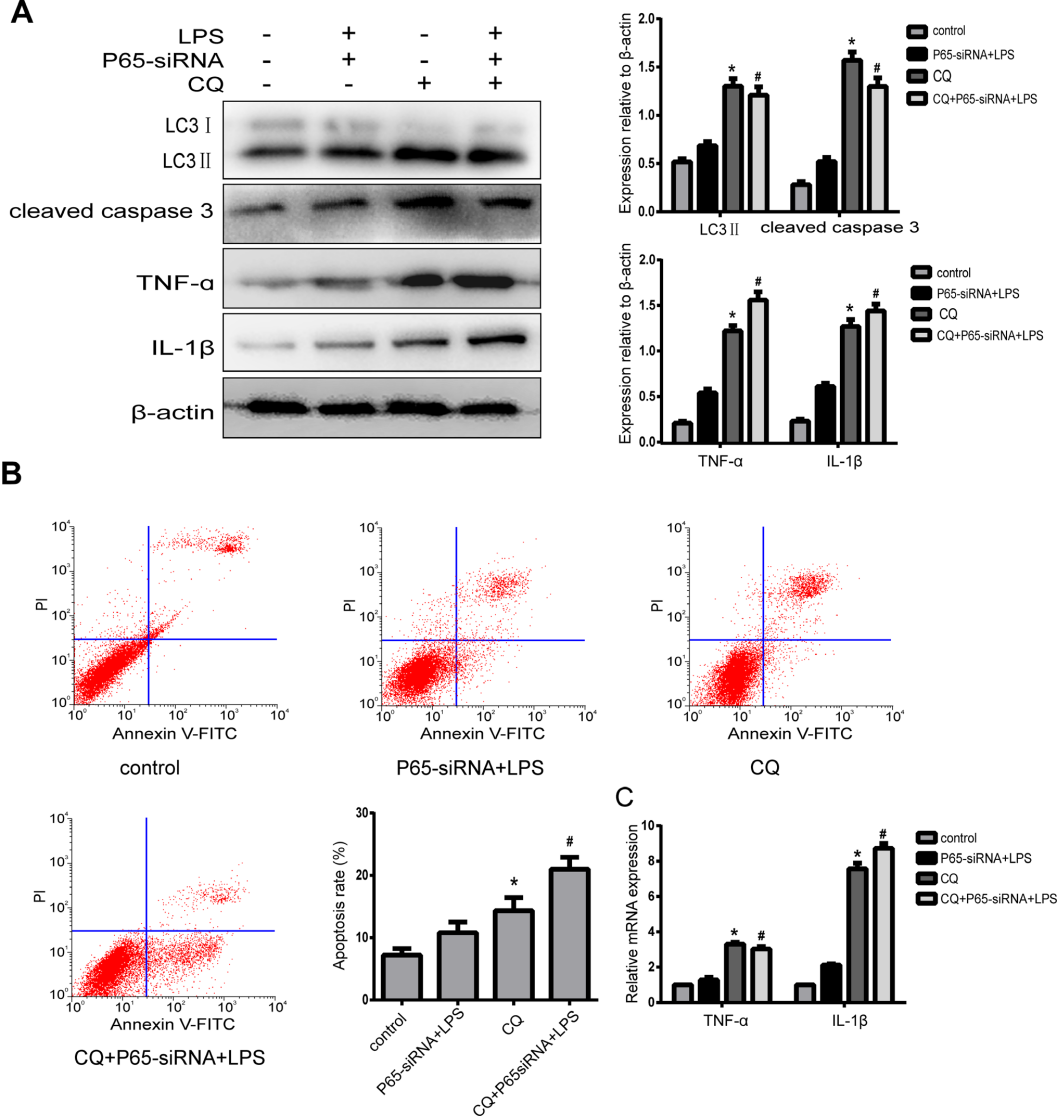
**Inhibiting NF-κB blocks apoptosis and inflammatory of degenerative human NP cells by promoting autophagy.** (**A**)After NPCs were pre-treated by autophagy inhibitor CQ, western blot analysis for the expression of LC3 II, cleaved caspase3, TNF-α and IL-1β. (**B**) The apoptosis rate was detected by flow cytometry. (**C**) Real-time analysis for the expression of TNF-α and IL-1β. Values are means ±SEM.*p<0.05 vs. control group, ^#^p<0.05 vs. P65-siRNA+LPS group.

In order to investigate whether the effect of NF-κB on NP cell apoptosis and inflammation was mediated by autophagy, the cells were pretreated with 10 um chloroquine (CQ), an autophagy inhibitor, which inhibits autophagosome-lysosome fusion. Western blot results revealed that the expression of LC3-II was significant higher in NP cells with pretreatment CQ than those treated with or without LPS+P65-siRNA, suggesting autophagy was inhibited. CQ pretreatment significantly increased the expression of cleaved caspase 3 and the incidence of NP cell apoptosis compared with or without LPS+P65-siRNA (Figure 5A and 5B). These results showed that NF-κB inhibition decreased NP cell apoptosis by promoting autophagy. Moreover, western blot and RT-PCR results showed that the protein and mRNA expression levels of TNF-α and IL-1β significantly increased in NP cells with CQ pretreatment compared with or without LPS+P65-siRNA, suggesting that NF-κB inhibition reduced NP cell inflammation by promoting autophagy (Figure 5A and 5C).

### Inhibiting NF-κB promotes autophagy through AKT/mTOR pathway

**Figure 6:**
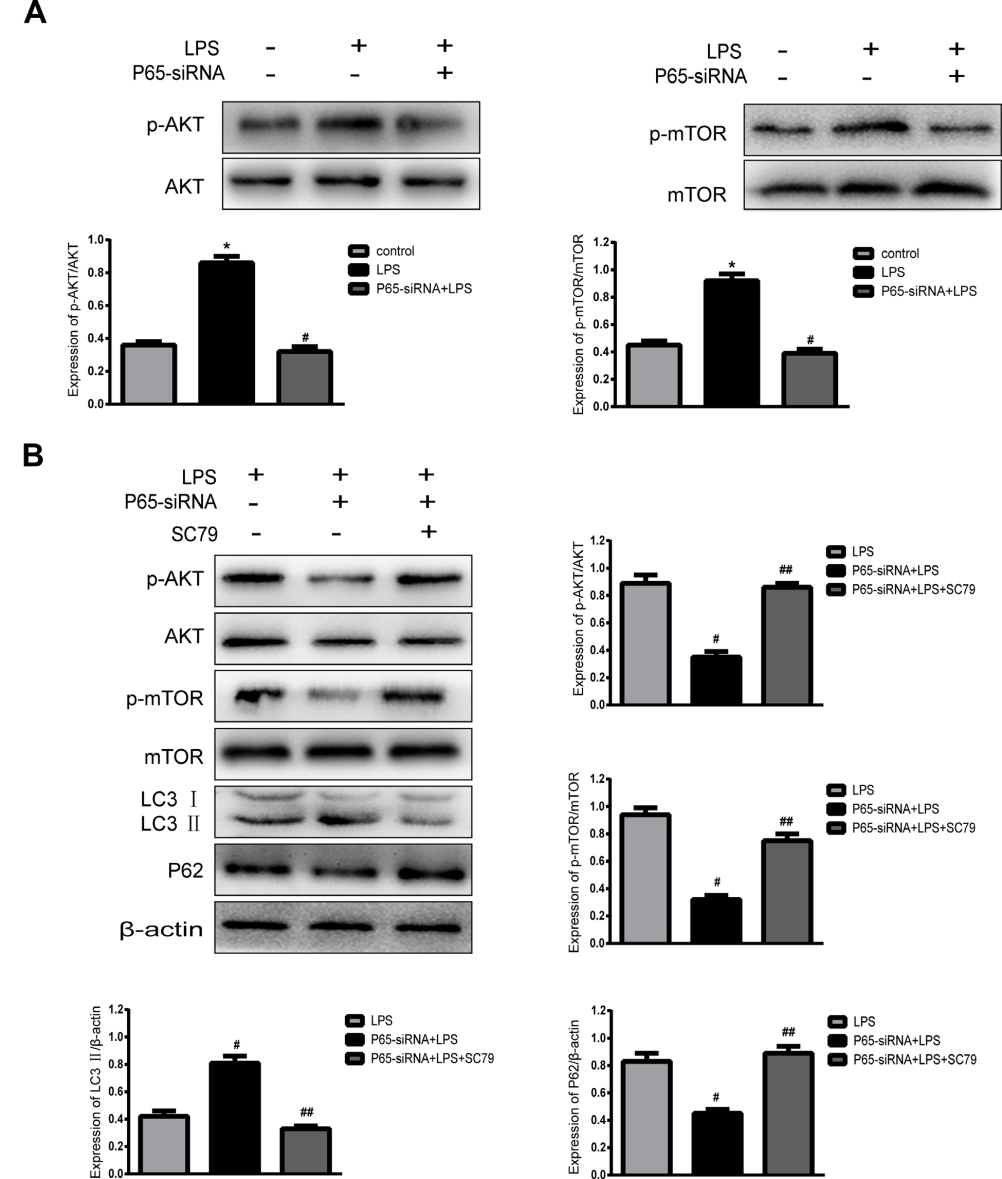
**Inhibiting NF-κB promotes autophagy through AKT/mTOR pathway.** (**A**) Western blot for the protein level of p-AKT, AKT, p-mTOR and mTOR. (**B**) After the addition of an AKT activator (SC79), Western blot for the protein level of p-AKT, AKT, p-mTOR, mTOR, LC3 and P62. Values are means ±SEM.*p<0.05 vs. control group, ^#^p<0.05 vs. LPS group, ^##^p<0.05 vs. P65-siRNA+LPS group.

In order to demonstrate the mechanism of inhibiting NF-κB to promote autophagy, the autophagy-related pathway proteins were measured by western blot. The results indicated that NF-κB inhibition significantly decreased p-AKT and p-mTOR expression compared with LPS treatment, while AKT activation (SC79) restored p-mTOR levels and decreased autophagy (Figure 6). In summary, Inhibiting NF-κB promotes autophagy through the regulation of the AKT/mTOR pathway in LPS- induced NP cells.

## DISCUSSION

In the present study, we demonstrate that knockdown of NF-κB can significantly reduce the apoptosis and inflammatory response in LPS-induced human nucleus pulposus cells, and promote autophagy through AKT/mTOR pathway. Furthermore, pharmacological inhibition of autophagy resulted in a marked increase in apoptosis and inflammation, indicating an anti-apoptotic and anti-inflammatory effect of NF-κB inhibition through induction of autophagy. These findings suggest there is a functional loop among apoptosis, inflammatory and autophagy in NP cells, in which NF-κB pathway seems to be a key.

Published evidence suggests that the pathological process of disc degeneration is closely related to the increase of oxidative stress, inflammatory cytokines, apoptosis and matrix metalloproteinases. Many studies have confirmed that proinflammatory factors can not only inhibit the synthesis of extracellular matrix, but also can induce NPCs apoptosis, leading to a decrease in the number of NPCs, thereby further aggravate the degeneration of the disc [5, 15]. Our present results showed that the expression of TNF-α, IL-1β and cleaved caspase 3 in degenerative disc disease (DDD) tissues are higher than those in lumbar vertebral fracture (LVF) tissues. Therefore, increased inflammatory factors and excessive apoptosis of nucleus pulposus cells may play a very important role in the process of disc degeneration. In addition, the P65 protein also increased in DDD tissues, indicating that the NF-κB pathway may be involved in the responses of inflammatory and apoptosis in IVDD. Consequently, it is necessary to explore the specific mechanism of the NF-κB pathway on the responses of inflammatory and apoptosis in IVDD.

NF-κB, a key nuclear transcription factor, plays an important role in initiating gene transcription of many cytokines, adhesion molecules and chemokines, and regulating inflammatory response, cell apoptosis and immune response. NF-κB is an upstream regulatory factor of inflammatory factors, Eren et al. found in lung injury, inhibiting NF-κB activity can inhibit the production of inflammatory factors [16]. In our present study, we also observe the same result in the human intervertebral disc: inhibiting NF-κB activity can significantly decrease the expression levels of TNF-α and IL-1β. In the degenerated intervertebral disc, high expression of TNF-α and IL-1β exacerbated proteoglycan degradation and NPCs apoptosis, which is related to NF-κB signaling pathway [17–18]. Jennewein et al. found that inhibiting of NF-κB activity can decrease the apoptosis of malignant glioma cells [11]. Jayasooriya et al. reported that activation of NF-κB promoted apoptosis through up-regulating Fas expression in human hepatocellular carcinoma cell line HepG2 [19]. Similarly, the same results were observed in our experimental results that knockdown of NF-κB significantly decreased apoptosis in LPS-induced NPCs. However, up to now, the specific mechanism by which NF-κB inhibition protects the nucleus pulposus cells from apoptosis and inflammation remains unclear.

Autophagy, a key adaptive response, is a conserved cellular process that can degrade and recycle intracellular damaged organelles and proteins [20]. It plays a critical role in the pathogenesis of degenerative diseases, such as osteoarthritis and Alzheimer’s disease [21–22]. Xu et al. found that autophagy attenuated the catabolic effect in rat nucleus pulposus cells, as sustained by NF-κB inhibition [14]. Thus, we speculated whether the NF-κB pathway and autophagy also had the same relationship in human nucleus pulposus cells. Our present results testified that inhibiting NF-κB activity promoted autophagy in human nucleus pulposus cells. Some previous studies have reported that autophagy played a protective role in the intervertebral disc [20, 23], but autophagy was a double-edged sword, adjusting to various stress [24]. Thus, to further confirm the relationship between apoptosis, inflammation and autophagy in our study, the autophagy inhibitor CQ was used to treat NPCs. Autophagy inhibition by CQ significantly increased cell apoptosis and inflammation, indicating that blocking the NF-κB pathway protected against apoptosis and inflammation by promoting autophagy. It has been reported that AKT/mTOR pathway was involved in cell autophagy [25–27] Simultaneously, the western blotting analysis directly indicated that after inhibition of NF-κB activity, the expression of p-AKT and p-mTOR protein markedly decreased, but the level of autophagy was inhibited after treatment with AKT activator SC79, suggesting the involvement of AKT/mTOR mediated autophagy in NP cells was under autophagy activation.

In summary, the present study revealed that a new mechanism associated with NF-κB inhibition attenuated impairment of cell inflammation and induction of apoptosis of LPS-induced NPCs. Knockdown of NF-κB promotes cell autophagy through AKT/mTOR pathway. In addition, autophagy triggered by NF-κB inhibition plays a protective role in LPS-induced NPCs. All these findings will contribute to a new direction for treatment of IVDD.

## MATERIALS AND METHODS

### Primary NPC isolation and culture

The collection and use of human IVD specimens were approved by an Ethics Committee of Chongqing Medical University before surgery. All samples were obtained in accordance with the World Medical Association Declaration of Helsinki Ethical Principles for Medical Research Involving Human Subjects. Written informed consent was obtained from all patients. All the samples were obtained from lumbar spine surgery patients. The control group was obtained from four patients with lumbar vertebral fracture (LVF), and the degenerative group was obtained from 15 patients with degenerative disc disease (DDD) during discectomy and intervertebral fusion surgery. The Pfirrmann classification was used to define degeneration grades of IVD by pre-operative MRI scans [28]. The relatively normal discs from LVF patients were defined as the Pfirrmann grade I or II, and the discs of all patients with DDD were defined as Grades III-IV.

The NP tissues from DDD patients were isolated immediately after surgery. Then the tissues were sequentially digested at 37 °C by 0.25% trypsin solution for 30 min and 0.2% type II collagenase for 4 hours. A 200-μm filter was used to remove tissue debris. The digested NP cells were cultured in DMEM/F12 medium (Gibco, USA) containing 10% FBS(Corning, USA) and 1% penicillin-streptomycin at 37 °C in a humidified atmosphere containing 5% CO2. The third generation of NPCs was used for all experiments.

### Cell treatment and transfection

The NP Cells were cultured with DMEM/F-12 medium with 2% FBS for 12 h before treatment and then were treated with 500 ug/ml LPS (Sigma, USA) for 24 h. The concentration of LPS was determined by the CCK-8 experiment in Figure 2. In addition, CQ (Sigma, USA) was used to analyze the autophagic flux and inhibit autophagy and AKT activator SC79 (MCE, USA), which were applied at 1 h prior drug treatment.

Negative control siRNA (NC-siRNA) and pre-designed siRNA against genes encoding NF-κB (P65-siRNA) were purchased from Santa Cruz Biotechnology (USA). Cells were transfected according to the manufacturer’s instructions.

### Immunohistochemistry

The NP tissues from four patients with lumbar vertebral fracture (LVF) and four patients with DDD were fixed in 4% paraformaldehyde for 48 h, and then were embedded in paraffin. The IHC staining procedure was performed through an immunohistochemical kit (Wuhan Boster Biological Technology, Ltd., Wuhan, China) according to the manufacturer’s protocol. Briefly, the tissues were incubated with trypsin for 30 min at 37 °C to retrieve the antigen. Next, the tissues were incubated with mouse anti-TNF-α and anti-IL-1β (Santa Cruz, USA), rabbit anti-cleaved caspase3 and anti-P65 primary antibodies (Cell Signaling Technology, USA) overnight at 4 °C. PBS was used as negative control. Then, the tissues were incubated with relatively secondary antibody IgG-HRP (155000) and counterstained with hematoxylin.

### Cell viability assay

The nucleus pulposus of logarithmic multiplication stage is digested by 0.25% trypsin, and then made into a single cell suspension. NPCs were prepared at a density of 1 × 10^4^/well in a 96-well plate and cultured for 24 h. The cells were subsequently treated with various concentrations of LPS (100, 200, 300, 400 and 500 μg/ml) for 24h, respectively. After treatment for 24 h, 10μl of the CCK-8 reagent was added to each well, and the cells were incubated for 2 h at 37 °C. The Bio-Rad 680 microplate reader (Bio-Rad, Hercules, CA, USA) was used to measure the absorbance of each well at 450 nm. Each experiment was performed in three replicate wells.

### Detection of apoptosis by Hoechst 33258 staining

NP cells were seeded in a 24-well plate at a density of 1×10^5^/well. After various treatment for 24 h, cells were fixed with 4% paraformaldehyde for 15 min and then washed with PBS. To observe the morphologic of apoptotic cells, 2 μg/ml Hoechst 33258 (Beyotime, China) was added to each well for 10 min at 37 °C. Apoptotic cells were characterized by showing brightly fluorescent DNA fragmentation and chromatin condensation under fluorescence microscopy.

### Detection of apoptosis by flow cytometry

Cells were cultured in six-well plates at 1 ×10^5^ cells per well. Apoptotic incidence was detected by using Annexin V/PI apoptosis detection kit (Keygen, China). Briefly, after different treatments for 24 h, NPCs were harvested and washed, and were suspended in binding buffer supplemented with 5 μl Annexin V and PI in the dark for 15 minutes at room temperature. The fluorescence intensities were measured by flow cytometry immediately. Apoptotic incidence including the percentage of early (Annexin V+/PI-) and late apoptotic cells (Annexin V+/PI+) were represented as a percentage of the total apoptotic cells.

### ELISA

After each group of cells was treated for 12 h, 24 h and 48 h, the supernatants were collected. The levels of TNF-α and IL-1β in cultured supernatants of NPCs were analyzed using ELISA kits according to the manufacturer’s instructions.

### Fluorescence immunocytochemistry

After each group of cells was treated for 24 h, Cells cultured in 24-well plates were washed with PBS several times and then fixed with 4% paraformaldehyde for 10 min. The cells were blocked with normal goat serum for 1 h, and then were incubated with P65 antibody (1:100, Cell Signaling Technology, USA)) at 4°C overnight. Finally, nuclear counterstaining was incubated with 4′6-diamidino-2-phenylindole. After they were washed three times with PBS, the cells were observed through a fluorescence microscope.

### GFP-LC3 analysis

To observe the autophagosomes, NP Cells were transfected with a GFP-LC3-expressing adenovirus for 24 h. Subsequently, the transfected cells were used for the next experiments. The autophagosomes were observed through laser confocal microscopy and the level of autophagy was evaluated with the number of green fluorescent puncta of autophagosomes.

### Transmission electron microscopy

After each group of cells was treated for 24 h, cells were collected and fixed in 2.5% glutaraldehyde. After dehydration, the samples were stained with uranyl acetate and lead citrate and then were observed by a TEM (Hitachi-7500, Japan).

### Western blot

Tissues and whole cell protein were extracted using RIPA lysis buffer, and then cell protein concentration was analyzed by BCA Protein Assay Kit (Beyotime, P0010S). After protein was transferred to PVDF membrane (0.45 or 0.22 mm), the membranes were blocked with 5% nonfat dry milk. Sequentially, the membranes were incubated with primary anti-IL-1β, TNF-α (Santa Cruz, USA), Bcl-2, Bax (OriGene, Herford, Germany), P65, LC3B, P62, p-AKT, AKT, p-mTOR and mTOR (Cell Signaling Technology, USA), β-actin (Beyotime, China) overnight at 4 °C. The membranes were incubated with the respective secondary antibodies for 2 h at room temperature. Finally, the membranes were measured by an ECL plus reagent (Millipore, USA) and the results were detected by the software.

### Real-time PCR analysis

The total RNA of human NP was extracted using TRIzol reagent (Invitrogen, USA) according to the manufacturer’s instructions. The expression of genes was determined by real-time PCR using ABI Prism 7500(ABI, USA) and SYBR® Green Real-Time PCR Master Mix (TOYOBO, QPK-201). The gene relative expression to the control was evaluated by 2-ΔΔCT method. The Primers for human genes as follows:

5′-GAAATGATGGCTTATTACAGTGGC-3′ and 5′-GC CACTGTAATAAGCCATCATTTC-3 for IL-1β; 5′-TC ATCTACTCCCAGGTCCTCTTCA-3′ and 5′-TGAAGA GGACCTGGGAGTAGAT- GA-3′ for TNF-α; 5′-GCAC CGTCAAGGCTGAGAAC-3′ and 5′-TGGTGAAGACG CCAGTGG- A-3′ for GAPDH; All the primers were synthesized by TaKaRa (TaKaRa, China).

### Statistical analysis

Statistical analysis was performed by one-way ANOVA or Student’s t-test using software SPSS 19.0. Data points from three independent experiments are expressed as the mean ± S.D. P value less than 0.05 was considered significant.
